# Nematodes in a polar desert reveal the relative role of biotic interactions in the coexistence of soil animals

**DOI:** 10.1038/s42003-018-0260-y

**Published:** 2019-02-15

**Authors:** Tancredi Caruso, Ian D. Hogg, Uffe N. Nielsen, Eric M. Bottos, Charles K. Lee, David W. Hopkins, S. Craig Cary, John E. Barrett, T. G. Allan Green, Bryan C. Storey, Diana H. Wall, Byron J. Adams

**Affiliations:** 1 School of Biological Sciences and Institute for Global Food Security, Queen’s University Belfast, Medical Biology Centre, 97 Lisburn Road, Belfast, BT9 7BL Northern Ireland UK; 2 International Centre for Terrestrial Antarctic Research, University of Waikato, Hamilton, 3240 New Zealand; 3 Canadian High Arctic Research Station, Polar Knowledge Canada, 1 Uvajuk Road, Cambridge Bay, NU X0B 0C0 Canada; 4 Hawkesbury Institute for the Environment, Western Sydney University, Penrith, 2751 NSW Australia; 5 Department of Biological Sciences, Thompson Rivers University, Kamloops, V2C 3A6 BC Canada; 6 SRUC - Scotland’s Rural College, West Mains Road, Edinburgh, EH9 3JG UK; 7 Department of Biological Sciences, Virginia Tech, Blacksburg, 24061 VA USA; 8 Gateway Antarctica, University of Canterbury, Christchurch, 8140 New Zealand; 9 Department of Biology, Colorado State University, Fort Collins, 80523 CO USA; 10 Department of Biology, Evolutionary Ecology Laboratories, and the Monte L. Bean Museum, Brigham Young University, Provo, UT 84602 USA

## Abstract

Abiotic factors are major determinants of soil animal distributions and their dominant role is pronounced in extreme ecosystems, with biotic interactions seemingly playing a minor role. We modelled co-occurrence and distribution of the three nematode species that dominate the soil food web of the McMurdo Dry Valleys (Antarctica). Abiotic factors, other biotic groups, and autocorrelation all contributed to structuring nematode species distributions. However, after removing their effects, we found that the presence of the most abundant nematode species greatly, and negatively, affected the probability of detecting one of the other two species. We observed similar patterns in relative abundances for two out of three pairs of species. Harsh abiotic conditions alone are insufficient to explain contemporary nematode distributions whereas the role of negative biotic interactions has been largely underestimated in soil. The future challenge is to understand how the effects of global change on biotic interactions will alter species coexistence.

## Abstract (web summary)

Tancredi Caruso et al. analyze biodiversity survey data from the McMurdo Dry Valleys, an extreme desert ecosystem in Antarctica in which abiotic factors are thought to determine species distributions. Focusing on three nematode species, they find that abiotic factors alone cannot explain the data and interaction between species have been historically underestimated.

## Introduction

The processes that structure biological communities have been investigated intensely in the last decades^1–4^ over a broad range of scales^5–8^. In recent years, the major goal has been to understand the relative roles of the different processes that structure ecological communities with a special emphasis on the balance between stochastic (e.g., dispersal dynamics) and deterministic (e.g., competition for shared resources, environmental filtering) processes^4,9–15^.

Despite much progress, most studies have focused on the aboveground component of terrestrial ecosystems. The belowground component has received far less attention but there have been studies addressing soil community structure at very broad scales^16–18^ as well as studies focusing on particular groups (e.g., bacteria, fungi, arthropods) at relatively local scales^6,19,20^: soil communities are structured at multiple spatial scales with local communities often consisting of species that are dispersal limited over relatively broad spatial scales^21,22^. Dispersal limitation and small population size of local and partially isolated communities imply that stochastic processes can interact with deterministic processes (e.g., the selection exerted by environmental variables) and contribute to community structure at multiple scales^6,22–25^.

One driver of soil community structure that remains poorly understood is biotic interactions. In aboveground communities, ecologists have been generating strong evidence that biotic interactions such as partitioning of resources within trophic levels play a fundamental role in structuring local communities^15,26,27^. In belowground communities, within trophic-group interactions have been shown to play a key role only for certain groups such as fungi^28^, whereas soil animals have been postulated to be less controlled by direct biotic interactions^22^. Still, soil is a very heterogeneous habitat even at fine spatial scales (e.g., < 100 cm) and it is well established that this heterogeneity promotes soil animal diversity^29^. Biotic interaction could structure soil animal communities via niche partitioning along environmental gradients^30^ as suggested by earlier investigators^31^. That is, species can spatially segregate and coexist at relatively broader scales (i.e., colonise different patches in the same landscape) if competition for resources is incompatible with their coexistence at local scales^12^. Falsifying this hypothesis as applied to soil ecosystems is very challenging because of the physical and chemical complexity of the soil habitat and its high biotic diversity, which includes aboveground–belowground linkages. To test for this, ideally one would need to control for all the abiotic, biotic, and historical factors (e.g., dispersal dynamics, biogeographical legacies, etc.) that affect the co-occurrence of species^32^.

To address this challenge in the present study, we targeted the soil ecosystem of the McMurdo Dry Valleys (MDVs) of Antarctica. These valleys represent the largest ice-free area of the Antarctic continent, host the driest and coldest soils on Earth and are part of the US National Science Foundation’s Long Term Ecological Research (LTER) network. The valleys support a simple soil community dominated by microbes and nematodes, both of which play major roles in regulating C and N cycling^33–35^ and coexist with a very few species of microarthropods, rotifers, and tardigrades. Species richness is indeed possibly the lowest on Earth and the harsh environmental conditions make it possible to identify the most important environmental determinants of species distribution (e.g., water availability)^35,36^. Thus, thanks to an inherently simple ecosystem structure, the MDVs offer a unique opportunity to test the role of biotic interactions in the assembly of soil communities. Current evidence suggests that the spatial distribution of the MDVs biota is driven by abiotic factors^37^. We aimed to quantify the role of biotic interactions relative to the roles of all the other factors we measured. We focused on the three most common species of nematodes that dominate the species-poor soil animal community. The three most common MDV nematode species are *Plectus murrayi, Eudorylaimus antarcticus*, and *Scottnema lindsayae*^38–41^, hereafter referred to by only their genus. Species from a fourth genus, *Geomonhystera antarcticola*, as well as another species of *Plectus*, *P. frigophilus*, are present but rare in the region^42^. The three common and dominant nematode species are known to have different environmental preferences and feeding strategies^43,44^. *Scottnema* is a generalist that feeds on bacteria and fungi and is found in high numbers in the extreme, dry, saline soils of the Dry Valleys^42,45,46^. Like *Scottnema, Plectus* is also bactivorous^47^, whereas *Eudorylaimus* is an omnivore that feeds on algae, cyanobacteria, and likely a variety of other metazoans^35,47,48^. In terms of their habitat preferences, *Plectus* and *Eudorylaimus* prefer higher levels of soil moisture than *Scottnema*^35,48^.

Given these feeding preferences and the the very high dispersal capability of these species, we hypothesised the following hierarchy of processes^15^: first, following dispersal, a certain species colonises an environmentally suitable patch, although this same patch can also be colonised or already inhabited by another species. Second, there are two possibilities: either feeding preferences of the two species do not overlap for at least one important resource (*Eudorylaimus*–*Plectus*; *Eudorylaimus*–*Scottnema*) or feeding preferences overlap (*Scottnema*–*Plectus*) for one important resource. If feeding preferences overlap, the two species compete for resources and might or might not coexist locally, depending on resource distribution. The outcome of competition will depend on abiotic conditions and resource supply/consumption, and each species will win different patches under different abiotic conditions or the two species could occasionally coexist in some patches. In any case, this process is expected to make species co-occur less often than expected by chance, that is, to segregate^32,49^.

In this study, we tested the null hypothesis that the three species of nematodes co-occur randomly. More specifically, we searched for evidence for the alternative hypothesis that *Scottnema* and *Plectus* may segregate/repel each other or be negatively correlated both because they have different abiotic requirements and/or interact negatively. Because *Eudorylaimus* could be preying upon *Scottnema* or *Plectus* (or both), we also tested the hypothesis that *Eudorylaimus* could be negatively correlated with these species. We found that abiotic factors alone are insufficient to explain contemporary nematode distributions and show that the role of negative biotic interactions has been largely underestimated in soil.

## Results

### Species distribution

*Scottnema*, *Eudorylaimus*, and *Plectus* were found, respectively, in 289, 222, and 50 out of 314 sampling points in which at least one nematode was found. At least two species co-occurred in 217 out of 314 locations. All three species co-occurred in only 30 locations. *Scottnema* and *Plectus* co-occurred 32 times out of the possible 50 points where *Plectus* (the less frequent species of the pair) was found. *Scottnema* and *Eudorylaimus* co-occurred 204 times out of the 222 possible times, and *Plectus* and *Eudorylaimus* co-occurred 41 times out of the 50 possible.

**Fig. 1:**
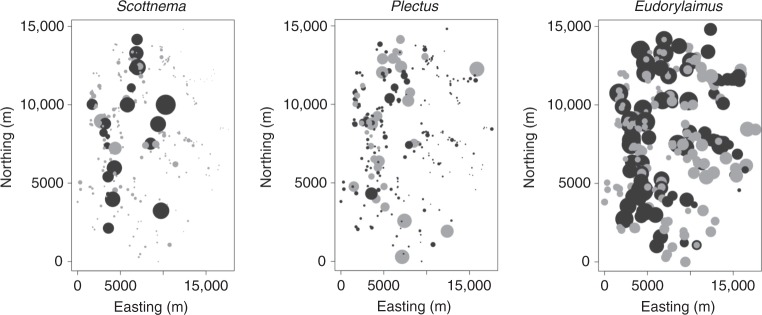
Map of the standardised residuals of *Scottnema*, *Plectus*, and *Eudorylaimus*. Residuals were obtained from a binomial GLM where predictors were the biotic and abiotic factors listed in Tables 1 and 2. Black and grey circles are negative and positive residuals, respectively. Negative correlation between *Scottnema* and *Plectus* is already evident in these maps and given these are residuals, this correlation does not depend on any measured environmental or biotic variables

The C-score, an index that quantifies checkerboard distributions, show that species co-occurred less often than expected by chance. The index value was, in fact, significantly larger than the central tendency of the null distribution (95% confidence limits: 663–752), corresponding to an effect size of 6.4. Bayes pair-wise analysis showed that two out of the three possible species pairs co-occurred non-randomly (*P* ≪ 0.001): *Scottnema* and *Plectus* segregated (effect size = 4.86), whereas *Scottnema* and *Eudorylaimus* aggregated (effect size = −4.32). Residuals from binomial generalized linear mixed models (GLMMs) (i.e., no modelling of spatial autocorrelation) displayed spatial structure when mapped (Fig. 1). This was particularly evident for *Scottnema* and *Plectus*. Autocorrelagrams based on Moran’s I confirmed there is spatial structure for neighbourhoods of about 4000–5000 m radius or less. GLMM for *Scottnema* revealed a significant (*P* < 0.05) negative effect of *Plectus*: the presence of *Plectus* significantly decreased the probability of occurrence of *Scottnema* while there is no effect of *Eudorylaimus* on *Scottnema* (Table 1). *Scottnema* was also positively correlated with the microbial biomass and richness gradient as well as the abundance of arthropods (Supplementary Figure 1). *Scottnema* was also negatively correlated to the soil moisture gradient, which correlated positively with electrical conductivity (i.e., the less water the higher the concentration of ions), pH, C, and N (Supplementary Figure 1). Also, *Scottnema* was positively correlated with the distance-to-the-coast gradient (Supplementary Figure 1). We observed a significant positive effect of the microbial biomass gradient and significant negative effect of *Scottnema* on Plectus (Table 2). *Eudorylaimus* (Supplementary Table 1) was not affected by the other two nematode species. Instead, the probability of occurrence of *Eudorylaimus* positively correlated with the richness of microbes and negatively correlated with the salinity and elevation gradient.

**Table 1:** Binomial GLMM of the probability of occurrence of *Scottnema*

	Estimate	S.E.	*t*-value	*P*-value
(Intercept)	3.846	0.561	4.901	**<0.001**
Microbial biomass gradient	−0.128	0.210	−0.521	0.544
Microbial richness gradient	0.500	0.234	2.213	**0.033**
Other fauna biomass gradient	0.065	0.204	−1.219	0.749
Organic matter gradient	−0.310	0.218	−1.925	0.157
Moisture gradient	−0.717	0.309	−2.410	**0.021**
Salinity gradient	0.132	0.247	1.557	0.593
Elevational gradient	0.403	0.379	1.256	0.290
Aspect factor	0.177	0.233	0.585	0.449
Distance to coast gradient	0.905	0.398	−3.023	**0.024**
*Eudorylaimus* (yes)	0.346	0.580	−0.606	0.551
*Plectus* (yes)	−2.224	0.592	−4.539	**<0.001**

Spatial autocorrelation in the residuals (Fig. 1) was modelled using a spherical function. The estimated parameters (Estimate) are in units of logit. See also supplementary results (a and b) and Supplementary Figure 1 for the measurement of biotic and abiotic gradients. *P*-values in bold are for *P*-value < 0.05

**Table 2:** Binomial GLMM of the probability of occurrence of *Plectus*

	Estimate	S.E.	*t*-value	*P*-value
(Intercept)	−1.376	0.701	–1.961	**<0.05**
Microbial biomass gradient	−0.747	0.174	–4.280	**<0.001**
Microbial richness gradient	−0.243	0.205	–1.180	0.238
Other fauna biomass gradient	−0.200	0.179	–1.118	0.264
Organic matter gradient	0.121	0.159	0.759	0.449
Moisture gradient	0.187	0.223	0.841	0.401
Salinity gradient	0.311	0.213	1.461	0.145
Elevational gradient	0.231	0.156	1.473	0.142
Aspect factor	0.065	0.217	0.299	0.765
Distance to coast gradient	−0.093	0.232	–0.390	0.691
*Eudorylaimus* (yes)	1.164	0.584	1.993	0.05
*Scottnema* (yes)	−2.254	0.630	3.578	**<0.001**

Spatial autocorrelation in the residuals (Fig. 1) was modelled using a spherical function. The estimated parameters (Estimate) are in units of logit. See also supplementary results (a and b) for the measurement of biotic and abiotic gradients. *P*-values in bold are for *P*-value < 0.05

### Multivariate analysis of species abundances

**Fig. 2:**
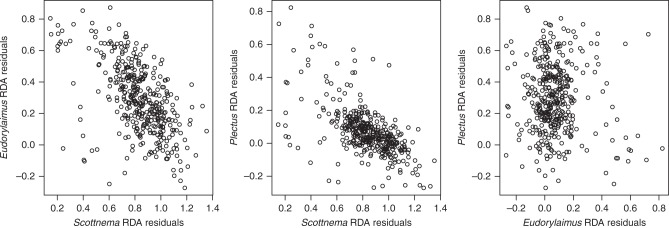
Scatter plots of the RDA residuals of *Scottnema*, *Plectus*, and *Eudorylaimus*. Residuals were obtained from an RDA model, the predictors of which were the same biotic and abiotic factors listed in Tables 1 and 2. RDA was applied to Hellinger transformed abundance data. Negative correlation is clear for the pairs *Scottnema*–*Plectus* (Pearson’s *r* = −0.59, *P* < 0.001) and the pairs *Scottnema*–*Eudorylaimus* (Pearson’s *r* = −0.60, *P* < 0.001)

A multivariate linear regression approach based on redundancy analysis (RDA) and variance partitioning showed that abiotic and biotic gradients plus spatial autocorrelation altogether account for 37% of total variance in the abundance of the three nematode species. Interestingly, spatial autocorrelation accounted for a relatively large and significant (*P* ≪ 0.05) fraction of variation (14 %) after controlling for the other factors, whereas biotic and abiotic factors accounted for 9% and 2% of variance, respectively. The remaining variance (12%) was shared among the three sources of variation. Residuals of the three species from the overall RDA model showed significant negative correlation (Fig. 2) for the pairs *Scottnema*-*Plectus* (Pearson’s *r* = −0.59, *P* < 0.001) and *Scottnema*-*Eudorylaimus* (Pearson’s *r* = −0.60, *P* < 0.001) no correlation was observed for *Plectus*-*Eudorylaimus* (Pearson’s *r* = −0.04, *P* = 0.49).

## Discussion

Negative biotic interactions such as competition for resources have been postulated to play a minor role in structuring soil animal communities^22^ despite suggestions by earlier investigators that partitioning of resources could explain the exceptional species richness observed in soil^31^. It is generally accepted that contemporary patterns of Antarctic biodiversity and ecosystem functioning are driven primarily by abiotic factors^50^. Thus, as pointed out by Convey et al.^51^, tests of hypotheses and model development for better understanding of patterns of species abundance and distributions must be dominated by measurements of abiotic environmental variables. The relative roles of biotic interactions has, however, remained unclear and the nzTABS project (see Methods) provided us with an optimal framework for developing models and testing hypotheses about these relative roles given its vast spatial scale and breadth and depth of biotic and abiotic variables measured. Our analysis shows, indeed, that a relatively large and statistically significant fraction of the variation observed in nematode distribution is spatially structured but independent of spatial variation in abiotic factors.

**Fig. 3:**
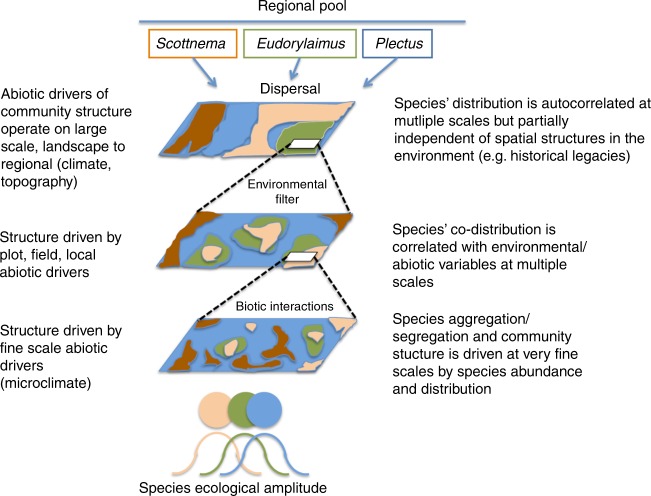
Conceptual model of the processes involved in the assembly of local belowground communities. The model reflects our modelling strategy. At large scales, dispersal determines whether a species colonises an environmentally suitable patch. Nematodes are not much dispersal limited. Environment is therefore filtering species from broader to smaller scales. In the patch, there might be local environmental heterogeneity but also interaction with other species, which either were already resident or may colonise the patch in a second moment. Species feeding preferences might not overlap (*Eudorylaimus*–*Plectus*; *Eudorylaimus*–*Scottnema*: see Gaussian curves of different colours), which makes local coexistence more probable. Or, species feeding preferences might overlap (*Scottnema*–*Plectus*), which might imply either local competitive exclusion and segregation or, in some cases local coexistence via stabilising mechanisms

This variation could be explained by biotic interactions, stochastic processes (such as dispersal), or a combination of both. The existence of a large fraction of spatial variance independent of variation in abiotic factors is consistent with our conceptual framework (Fig. 3)^12,15^. In fact, in belowground communities part of this variation can be generated by the dispersal processes that link species regional pools to local communities^19,21^. These processes may well operate in terrestrial Antarctic ecosystems as inferred also by other authors^25,52^. Processes other than dispersal dynamics can, however, contribute to the fraction of spatial variation that is independent of abiotic factors and this might be particularly true for nematodes. Nematodes in the MDVs are not dispersal limited thanks to their life history and survival strategies, particularly cryoprotective dehydration^46,53^. Source-sink dynamics may thus support species in unfavourable patches, where conditions would usually cause species extinction after initial colonisation. High dispersal rates of nematodes can indeed support this dynamic, which is known as “mass effect” in metacommunity theory.

Once a given species reaches a certain patch, abiotic and biotic conditions will determine whether or not the colonisation is successful (Fig. 1). This selection process is expected to create patterns of covariation in species distribution and abiotic/biotic conditions^12,54^. Two out of the three investigated species clearly showed this covariation and multivariate analysis showed that a significant fraction of variation in the overall assemblage was accounted for by abiotic factors. *Scottnema* is well adapted to dry, saline soil and known to be frequent and abundant at high elevations^44,53^. Our *Scottnema* GLMM confirms this. *Eudorylaimus*, too, is known to be well adapted to the Antarctic environment. However, studies of its ecology have highlighted that this species prefers moister conditions and relatively lower salinity than *Scottnema*, which might also be due to the fact that the Antarctic species of this genus can feed on algae^35,44^. Our *Eudorylaimus* GLMM is consistent with this notion. Also, the multivariate analysis demonstrates that part of the negative covariation between these two species can be due to their opposing responses to environmental gradients.

Abiotic factors thus act on two fundamental gradients in our system. The first is elevation, which together with distance to the coast affects temperature and water availability. The second is salinity, which is moderated by local topography but also partially depends on elevation and distance to the coast. *Plectus* was by far the least frequent and abundant of the three species, which is consistent with previous studies^55^. This species is typically associated with high moisture and its distribution is limited by the generally low soil moisture conditions of the MDVs^42^. Nevertheless, there are several patches where this species co-occurs with the other two species, as well as patches where *Plectus* was the only species present. Therefore, there are specific locations where *Plectus* can establish populations, at least in the short term, and perform even better than the other two species.

Two or more species often co-occurred in the same patch, and often at high densities. *Eudorylaimus* and *Scottnema* aggregated even though their relative abundances were negatively correlated. In general, the fact that the three species co-occur in a relatively high number of samples implies that these species could reach the same patch and cope with the abiotic and biotic conditions of this patch; *Eudorylaimus* might feed on a variety of food items (omnivorous), thus possibly avoiding competition with *Plectus* and *Scottnema* and potentially be indirectly favoured by C-cycling functions performed by *Scottnema*. Also, we cannot exclude the possibility that *Eudorylaimus* feed, at least occasionally, on *Plectus* and *Scottnema*, which could explain the negative correlation between the relative abundances of *Scottnema* and *Eudorylaimus*.

Our results suggest that *Plectus* and *Scottnema* could potentially compete for bacteria. That both these species feed on bacteria is a well established known factor, and our models show that bacterial richness and biomass positively correlate with frequency and abundance of both species. *Plectus* most likely feeds only on bacteria, whereas *Scottnema* is thought to feed on bacteria and yeast^35,53^. Under the hypothesis that these two species compete for one resource, our data empirically show that the two species have an ability to coexist locally under certain conditions. Thus, we can postulate the existence of stabilising niche differences that allow coexistence under specific conditions^15^. However, in most cases the two species do not co-occur and the analyses we have performed clearly suggest that the two species spatially segregate. This segregation cannot be fully explained by the numerous biotic and abiotic variables measured in the study. Given background knowledge and considering all the other factors taken into account in our statistical models, the observed pattern of segregation can be interpreted parsimoniously as a relative fitness difference^15^. When two species compete for a resource, relative fitness differences make one of the two species the best competitor for the resource (in this case bacteria) under a given set of abiotic and biotic conditions. However, if the conditions change, the other species can become the best competitor. Indeed, our modelling suggests that the two species respond to abiotic conditions differently: *Scottnema* is adapted to high salinity and low moisture and tolerate conditions under which *Plectus* would not survive (with or without competition). *Plectus* dominates only when soil moisture is much higher and salinity is lower than the average levels of MDVs. The two species have substantially different life strategies as *Scottnema* is stress tolerant and can survive in most MDV soil, being also able to grow optimally at low temperatures (optimum at ~10 C°)^45^, whereas *Plectus* flourishes at higher temperatures^56^.

In the past, biotic interactions have been considered a minor determinant of Antarctic species distribution in the belowground because there is little if any top down control (e.g., predators of microbial grazers are generally absent); and fundamental abiotic conditions such as availability of liquid water are extremely limiting^37^. More generally, the role of biotic interactions, especially competition, in determining distribution and coexistence of belowground invertebrates has been downplayed although not ruled out^22,57^. In this work, for the first time, we have shown that negative covariation between species of nematodes can be partly explained in terms of negative biotic interactions, besides the well-known effects of a range of abiotic factors, such as soil moisture and salinity. Future manipulative experiments will, however, have to directly test for these interactions explicitly.

The first implication of our findings is that biotic interactions could simply be underestimated in soil ecosystems because their effects are difficult to detect in the field and entangled with the effects of other, more macroscopic abiotic factors (e.g., large-scale gradients in salinity and moisture)^27^. Second, conditions for coexistence are more restrictive locally than globally^30^ as many species cannot always coexist locally but can colonise and win different patches in the same landscape, thereby still coexisting at broad scales if not at local scales. The interaction between the selection exerted by abiotic factors and biotic factors is expected to affect ecosystem functioning. In the case of the MDVs, *Scottnema* plays a crucial role in cycling soil organic C^34^. At the same time, climatic changes are happening at increasing rates in some areas of Antarctica^58^. These changes are drastically and negatively affecting the abundance and distribution of *Scottnema*^34,58^. We thus expect biotic interactions between *Scottnema* and other species to be altered by climatic changes because in many areas abiotic conditions are becoming less favourable to *Scottnema*. These changes have a great potential to affect ecosystem functioning^35^, in particular nutrient cycling, at larger scales^34^. More generally, our findings imply that the abiotic harshness of extreme environments alone is insufficient to explain the distribution of species across the landscape. Thus, biotic interactions may play a role in community assembly and subsequent ecosystem structure and functioning heretofore underestimated. The future challenge therefore is to quantify how environmental changes interfere with biotic interactions and affect the local and global coexistence of species and thus the effects of animal communities on ecosystem functions.

## Methods

### Study area and sampling design

**Fig. 4:**
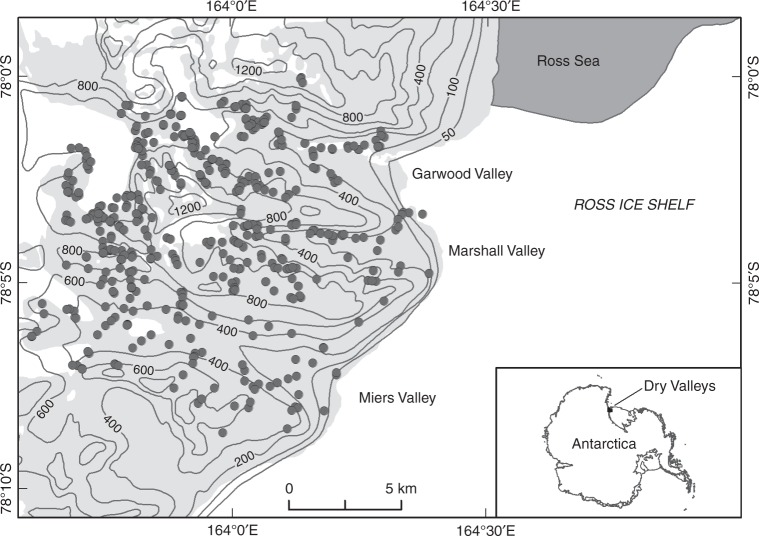
Map of the 220 km^2^ study area, in the McMurdo Dry Valleys. The area was divided into 554 geographically and geologically distinct ice-free sectors using remote-sensing data, and with geographical and geological variables validated on-the-ground assessments in November 2008. Eventually, 471 sites were sampled in January 2009 and January 2010 to encompass the entire range of geographical and geological heterogeneity in the area. Nematodes were found in 314 sites

This study was conducted in the context of the New Zealand Terrestrial Antarctic Biocomplexity Survey (nzTABS, http://nztabs.ictar.aq), which was initiated during the International Polar Year 2007–2008, and drew a diverse range of international expertise to profile the biology, geochemistry, geology, and climate of the Dry Valleys (Fig. 4). The study is among the most comprehensive landscape-scale biodiversity surveys undertaken and includes nearly all trophic components found in the Dry Valley ecosystem. A Geographic Information System (GIS) model including geological and geomorphological information and augmented by analyses of ALOS, LandSat, and MODIS satellite imagery, aerial photographs, and subsequent field mapping was used to divide the 220 km^2^ study area into 554 geographically and geologically distinct ice-free sectors (minimum area of 1.5 km^2^) referred to as landscape tiles. Tile boundaries were delineated where the combination of geographical and geological variables changed, which on average happened over a few km. On-the-ground environmental assessments were carried out in November 2008 to confirm the reliability of delineations. A total of 471 sites were chosen to encompass the entire range of geographical and geological heterogeneity in the sampling campaign. Sampling of soils and biological communities was carried out over two successive Austral summers (January 2009 and January 2010).

### Sampling abiotic and biotic variables

At each sample site, the top 10 cm of soil was collected using sterile techniques with a trowel from multiple spots within a 1 m^2^ area for the following subsamples: bulk soil (~400 g) with large pebbles ( > 2 cm diameter) removed aseptically and homogenised in a sterile 42 oz Whirl-Pak® bag; soil (~20 g) for moisture content measurement, subsampled from homogenised bulk soil into a sterile 15 mL centrifuge tube sealed with Parafilm®; soil (~300 g) for analysis of metazoans, stored in a sterile 18 oz Whirl-Pak® bag. The samples were staged in coolers in the field at ambient temperatures for up to 72 h prior to transport to the laboratory for further analysis.

Total soil moisture content was determined gravimetrically by the mass loss of soil heated to 105 °C for 48 h and recorded as percentage moisture content. For total organic carbon and total organic nitrogen contents, soil was air dried and ground in a ball mill to a fine homogenous powder. A 300 mg acidified aliquot of the homogenised soil was then analysed using a CE Elantech Flash EA 1112 Elemental Analyzer (Lakewood, NJ) at the Virginia Tech Ecosystem Analysis Laboratory^59^. Soil pH and conductivity were measured using the slurry method^60^. In brief, 10 mL of deionised water was added to a soil aliquot (2 mL) and mixed thoroughly. The pH and conductivity of the resulting slurry was measured using a Thermo Scientific Orion 4-Star Plus pH/Conductivity Meter (Thermo Scientific, Auckland, NZ).

A number of key environmental attributes were derived from satellite imagery and the custom digital elevation model created for the project, including basic topographic variables (such as elevation, slope, and aspect), surface soil temperature, a topographically derived “wetness index”, and distance to the coast. Soil surface temperatures were obtained from Landsat 7 ETM+ using band 6 at 60 m resolution, which captured the up-welling thermal infrared spectrum (in the 10.4–12.5 μm band). Replicated, summer Landsat 7-derived temperature data corresponding to locations of 45 on-the-ground temperature loggers (DS1921G iButtons, Maxim Integrated, San Jose, CA) were compared with records from the iButtons, and significant positive correlations between the two data sets were found^61^.

Nematodes, tardigrades, and rotifers were extracted from soils using the modified sugar centrifugation technique (see Supporting Information for details). Individual animals were identified and enumerated (both live and dead) using bright-field microscopy (Olympus CK40 Inverted Microscope, Olympus America Inc., Center Valley, PA). Nematodes were further identified into species, gender, and life stage (adult, juvenile) using Timm, Andrássy, and Boström et al.^38–41^. Population abundances were recorded as numbers of individuals per kg soil, corrected to oven-dry weight equivalent. The presence and abundance of flagellates, amoebae, and ciliates was also recorded. However, the data were not included in the analysis since reliable characterisations of protozoan abundance and diversity exceeded our logistical capability^62^.

Microarthropods (Acari and Collembola) were collected from the underside of small, flat, rocks within a 20 m radius of where the soil sample was taken. Individual animals were collected using an aspirator and preserved in 100% EtOH, enumerated, and identified using the approach described in^63^.

Total environmental DNA was extracted from soil samples using a CTAB protocol^60^ modified for the X-tractor Gene liquid handling robot (Corbett Life Sciences, Concorde, NSW, Australia). See Supplementary Methods for further details. The extracted environmental DNA was quality-checked and quantified as a surrogate for microbial biomass using Quant-iT Picogreen dsDNA reagent (Invitrogen, Auckland, New Zealand) on a FLUOstar optima fluorescence plate reader (BMG Laboratories, Offenburg, Germany. See Supplementary Methods for more details). Diversity of bacteria and fungi was estimated by automated ribosomal intergenic spacer analysis (ARISA^64^; see Supporting methods *b* in the Supporting Information document for details).

Soil respiration was measured by incubating 20 g (dry weight equivalent) samples of soils at 10 °C for 26–28 days in an miniaturised respiromotric chambers^65^ and the CO_2_ determined periodically by gas chemotography (Varion90 GC fitted with a thermal conductivity detector) as described by^66^.

### Modelling

To collect evidence for our hypothesis that negative biotic interactions can structure the co-occurrence of the three species of nematodes, we first tested the general null hypothesis that species co-occur randomly (see Supporting Methods, Supplementary Figure 2). First, we used the species presence/absence matrix to calculate the C-score, an index that quantifies checkerboard distributions: species that do not co-occur very often produce a high index value and vice versa^67^. Second, we applied null model analysis^49^ to the C-score by using a randomisation scheme that preserved row and column totals (algorithm SIM9 in Gotelli^49^). This is the most effective randomisation approach to isolate non-random patterns caused by biological interactions because it randomises only species composition. The C-score plus the randomisation scheme we employed has been shown to lower the risk of false positives while maintaining good statistical power^49^. The null distribution of the C-score was obtained from 5000 random matrices. The central tendency of the null distribution was then compared with the observed C-score. The C-score was also calculated on a species-pair basis and tested following the method of Gotelli and Ulrich^68^ and the Fortran program Pairs^69^: this method builds confidence limits using the empirical Bayes approach. In null models, effect size was calculated as $\frac{{ {\rm obs}. {\rm index} - {\rm exp}. {\rm index}}}{{ {\rm null}\, {\rm S.D.}}}$, where obs.index is the observed C-score, exp.index is the central tendency in the C-score null distribution, and null S.D. is the Standard Deviation of the C-score null distribution.

To isolate the relative effects of abiotic and biotic variables, we modelled the effect of the presence of one species on the other while statistically controlling for all other measured variables. We thus analysed the presence/absence distribution of each species using GLMM (spatial generalised linear mixed model^70^); with binomial error structure^71,72^. These models estimate the probability that a particular species colonises a certain patch and how measured abiotic and biotic variables together with the presence of the other two species of nematodes affected this probability. In order to avoid collinearity between predictors and model overfitting (i.e., too many and too correlated predictors), we applied principal component analysis (PCA) to the correlation matrix of abiotic and biotic variables to estimate major gradients in the system. We selected the first PCA axes that accounted for at least 2/3 of total variance^73^ and used these axes to quantify major abiotic and biotic gradients (see Supplementary Methods and Supplementary Figure 1 for details on how biotic and abiotic predictors were introduced in the models). GLMM models also allowed us to quantify the size of the effect of each variable on the occurrence of each species while controlling for spatial autocorrelation in species distribution. We used a spherical autocorrelation function^74,75^ to model spatial autocorrelation.

Recent studies have shown that co-occurrence data might reflect biotic interactions only partially^76,77^ and we thus completed the analysis with a multivariate modelling of the relative abundances of species. Specifically, we applied Hellinger transformation to the matrix of abundance of the three nematode species. This allowed us to correctly apply Redundancy Analysis, a multivariate form of linear regression^73^, to species abundance data^78^. The predictors used in RDA were the same as the predictors used in GLMMs. In addition, we used principal coordinate analysis of neighbour matrices (PCNM^79^); to account for spatial autocorrelation. Following^80^, we used a multivariate extension of the Akaike Information Criterion (AIC) to select the most parsimonious set of vectors accounting for the largest amount of autocorrelation in species distribution. Variance partitioning was calculated to quantify the amount of variation accounted for by each set of predictors (abiotic, biotic variables and PCNM eigenvectors, below called “spatial vectors”). Finally, RDA residuals of each of the three nematode species were correlated to the residuals of the other two species. GLMMs and RDA were run in R version 2.15.1^81^ using the package MASS^70^ and vegan^82^.

### Code availability

R codes used for Multivariate modelling and GLMM models are available as Supplementary Software 1 and 2.

## Supplementary information

Supplementary Information

Supplementary Data 1

Supplementary Software 1

Supplementary Software 2

Description of Additional Supplementary Files

## Data Availability

The full dataset is uploaded online as Supplementary Data 1.
