# Supplementary material for: Nematodes in a polar desert reveal the relative role of biotic interactions in the coexistence of soil animals
Source: Commun Biol. 2019 Feb 15;2:63. doi: 10.1038/s42003-018-0260-y (PMC6377602; doi:10.1038/s42003-018-0260-y)
Supplement: Supplementary file 1 — Supplementary Information [file 42003_2018_260_MOESM1_ESM.pdf]

Supplementary Information

Supplementary Figures

Supplementary Figure 1

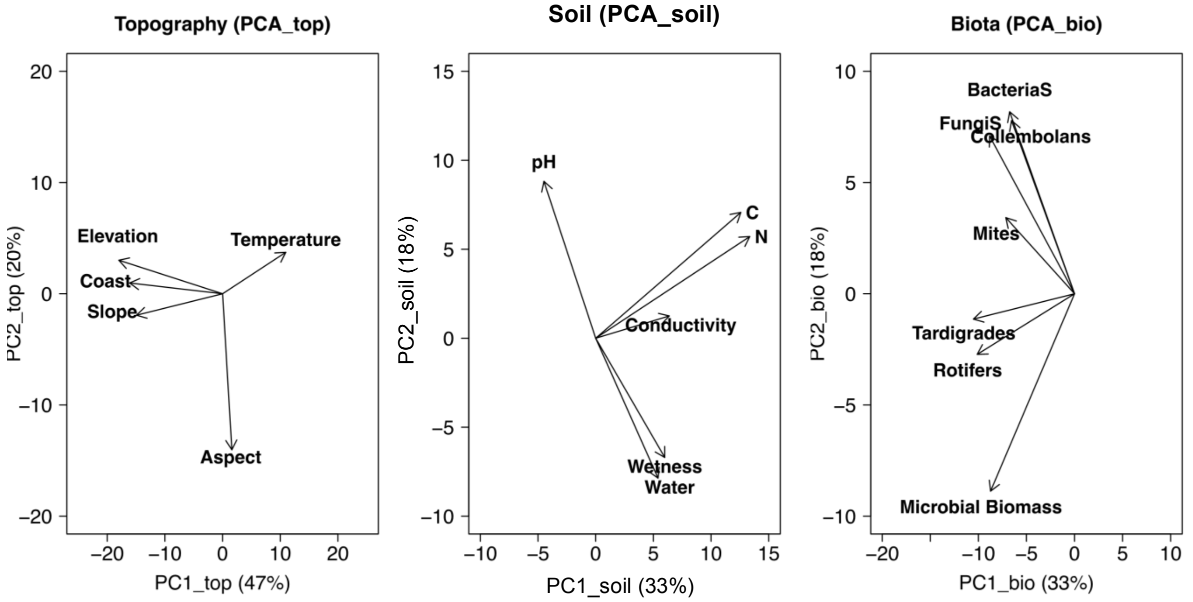

Topography and Soil (abiotic factors), and other biota (biotic factors): PCA was applied to the three correlation matrices that collected information on these three sets of variables. The first two axes of each of the three PCAs are plotted together with the vector variables. The length and direction of vectors is a function of the loading of variables on the first two PCA axes. Ordered observations (i.e. data points) are not shown. Some of the vector labels are clarified below (the others are self-explanatory): “Coast”, distance to the coast; “Conductivity”, electrical conductivity; “C”, organic carbon; “N”, total nitrogen. The vector of Cyanobacteria richness is not plotted because it mostly overlapped with that of Bacteria richness. For the same reason, only microbial biomass and not respiration were plotted.

Supplementary Figure 2

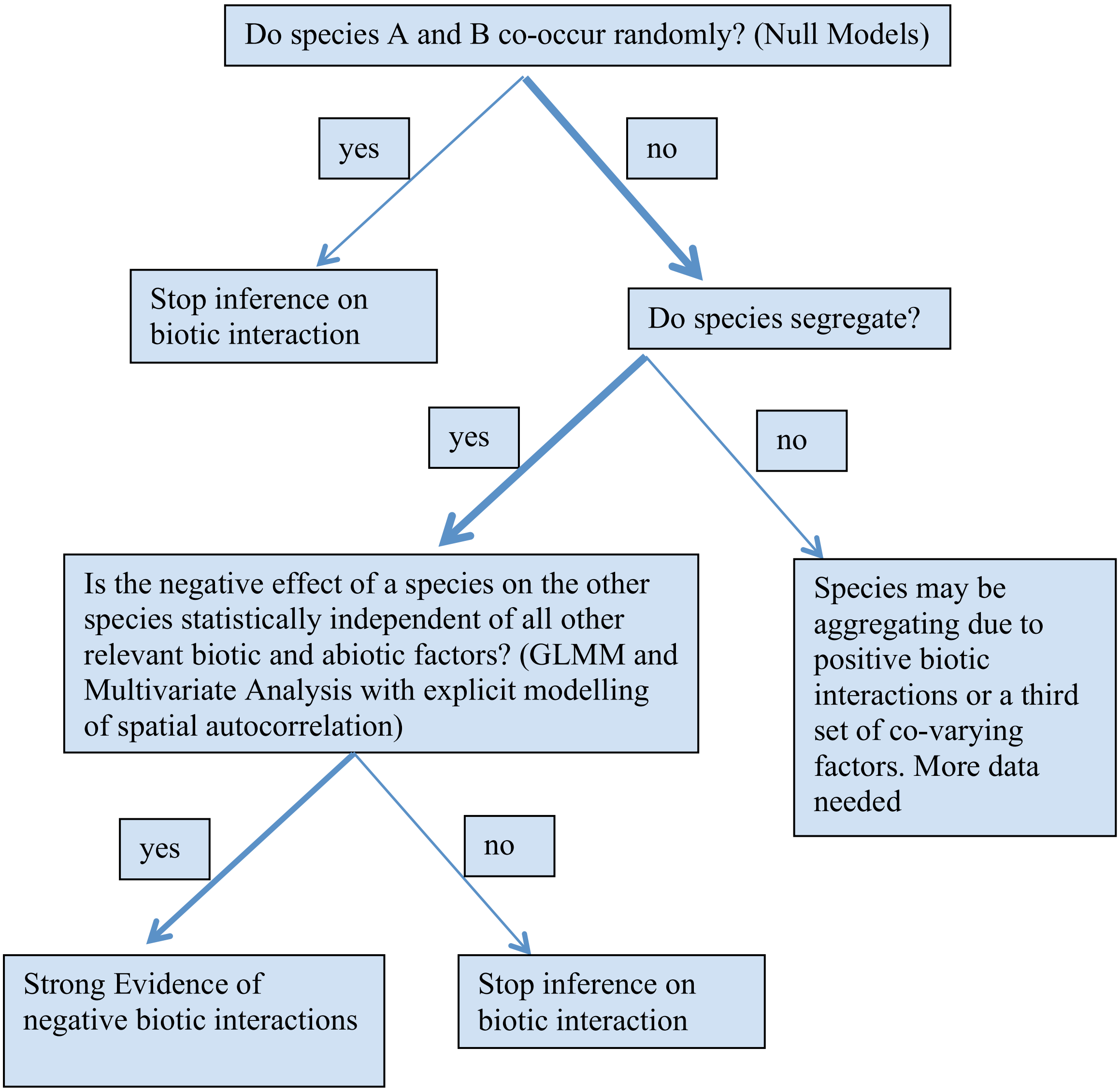

Statistical evidence for negative biotic interactions requires three bits of evidence (Solid, thicker blue arrows): 1, verification that species co-occur non-randomly and, specifically, that they segregate; 2, statistical control of all other biotic and abiotic factors; 3, modelling of spatial autocorrelation.

Supplementary Tables

Supplementary Table 1

Binomial GLMM of the probability of occurrence of *Eudorylaimus*. Spatial autocorrelation in the residuals (Figure 2) was modeled using an exponential function. The estimated parameters (Estimate) are in units of logit

	Estimate	S.E.	t-value	p-value
(Intercept)	0.552	0.549	1.007	0.315
Microbial biomass gradient	-0.185	0.106	-1.738	0.083
Microbial richness gradient	0.638	0.128	4.968	**<0.001**
Other fauna biomass gradient	-0.003	0.137	-0.024	0.981
Organic matter gradient	-0.087	0.099	-0.870	0.385
Moisture gradient	0.000	0.138	-0.003	0.997
Salinity gradient	-0.311	0.124	-2.510	**0.013**
Elevational Gradient	-0.243	0.086	-2.839	**<0.001**
Aspect factor	-0.124	0.134	-0.929	0.354
Distance to coast gradient	-0.132	0.141	-0.933	0.351
Scottnema present	0.305	0.557	0.548	0.584
Plectus present	0.859	0.504	1.703	0.089

Supplementary Methods

Extraction of Fauna

Nematodes, tardigrades and rotifers were extracted from soils using the modified sugar-centrifugation technique of [1]. In this method, fauna in soil are mixed with water and poured through stacked 500 and 38µm sieves to remove large rocks and debris. The resulting slurry is collected in a clean tube, centrifuged to separate the animals and soil from the water, and the supernatant removed. The pellet of animals and soil are then remixed in a dense sugar solution that is again briefly centrifuged. The soil and debris form a pellet and the animals are contained in the supernatant. The supernatant is washed over a 26µm sieve and specimens are collected, and stored in water. The animals are moved to counting dishes and identified using an inverted compound microscope. An advantage of the method is that it allows the collection of both living and dead animals. Nematodes were identified to species level [2–5]. Tardigrades and Rotifers were identified no further than phylum.

Soil microarthropds in Antarctica live in the first few cm of soil and indeed mostly between rocks and the substrate. Thus, the best method to represent their fauna if collecting them from the underside of small, flat, rocks [6–8]. In order to have quantitative estimates of their densities, specimens were collected within a 20m radius of where the soil sample for mesofauna was taken. Identification of specimens following the basic descriptions of [9] and follow up publications: see the general review by [7] for a full list of taxonomic references.

Molecular Methods

Total environmental DNA was extracted from soil samples using a CTAB protocol [10] modified for the X-tractor Gene liquid handling robot (Corbett Life Sciences, Concorde, NSW, Australia). Briefly, 0.7 g of soil was added to a microcentrifuge tube containing 0.5 g each of 0.1 mm and 2.5 mm silica-zirconia beads (BioSpec Products, Bartlesville, OK, USA), 270 µL phosphate buffer (100 mM NaH_2PO_4), and 270 µL SDS lysis buffer (100 mM NaCl, 500 mM Tris pH 8.0, and 10% SDS). Samples were bead-beaten for 10 minutes on a Vortex Genie 2 with a 24-tube vortex adapter (Mo Bio Laboratories Inc., Carlsbad, CA, USA). 180 µL CTAB extraction buffer (100 mM Tris-HCl, 1.4 M NaCl, 20 mM EDTA, 2% CTAB, 1% PVP, 0.4% BME) was then added, and samples were shaken at 300 rpm and 60 °C for 30 minutes. Samples were centrifuged at 16,000 g for 3 minutes, prior to the addition of 350 µL chloroform:isoamyl alcohol (24:1) and 35 µL 10 M ammonium acetate. Samples were vortexed and centrifuged at 16,000 g for 5 minutes. A 200 µL aliquot of the aqueous phase of each sample was transferred to a 96 well lysis block and processed using an X-tractor Gene liquid handling robot (Corbett Life Sciences, Concorde, NSW, Australia), using the DX Universal liquid sample DNA Extraction Protocol (CorProtocol No. 14104 Version 02). Samples were eluted in 80 µL TE pH 8.5 (10 mM Tris-HCl, 0.5 mM EDTA). Negative controls, consisting of bead tubes with no sample added, were processed as described above and included in each lane of the lysis block to assess potential contamination of extracts. For samples yielding DNA concentrations less than 1.8 ng/µL, extractions were repeated manually, without processing on the X-tractor Gene, to increase yields. The lysis steps were completed as outlined above, and the method after the chloroform step was modified as follows. The final ammonium acetate concentration of the lysate was brought to 2.5 M, 300 µL chloroform:isoamyl alcohol (24:1) was added, samples were vortexed, and centrifuged at 16,000 g for 5 minutes. The entire aqueous phase was transferred to a new tube and the chloroform step repeated with an equal volume of chloroform:isoamyl alcohol (24:1). The

aqueous phase was transferred to a new tube and DNA was precipitated with addition of 0.54 volumes of isopropanol followed by centrifugation at 16,000 g for 20 min. Pellets were washed by adding 1 mL 70 % ethanol, centrifuged at 16,000 g for 5 min, and the supernatant discarded. Dried pellets were resuspended in 30 μL TE pH 8.5.

The extracted environmental DNA was quality-checked and quantified as a surrogate for microbial biomass using Quant-iT Picogreen dsDNA reagent (Invitrogen, Auckland, New Zealand) on a FLUOstar optima fluorescence plate reader (BMG Laboratories, Offenburg, Germany). Briefly, 100 μL of picogreen solution (picogreen diluted 1:200 in TE) was added to each well of a black 96 well plate, containing 95 μL TE and 5 μL sample or standard containing 0 to 25 ng/μL lambda dsDNA (Invitrogen). Samples were excited at 485 nm and emission was measured at 520 nm. All extracts with DNA concentrations exceeding 2.2 ng/μL, were adjusted to between 1.8 and 2.2 ng/μL in TE to ensure consistent template concentrations in community analyses. DNA was below limits of detection in all negative controls.

Another surrogate for microbial biomass, total soil ATP, was measured in duplicates (triplicates where the duplicates did not match) using a 3M Clean-Trace Beverage Test Kit (Acorn Scientific, Auckland, NZ) with a modified protocol. In short, 100 μL of Extractant Buffer was added to 100 mg of soil and allowed to incubate for 60 seconds. 75 μL of ATP Assay Solution was then added to the sample, which was immediately read using a 3M Clean-Trace NG Luminometer (Acorn Scientific). Total soil ATP levels were recorded as relative fluorescence units, and pure ATP solutions were used to check for signs of inhibition in samples with low readings. Diversity of bacteria and fungi was estimated by automated ribosomal intergenic spacer analysis (ARISA).

Diversity of bacteria and fungi was estimated by automated ribosomal intergenic spacer analysis (ARISA). Briefly, PCR targeting the ribosomal intergenic spacer of the bacterial and

fungal ribosomal operon was carried out for all extracted DNA samples and procedural negative controls using the following primer pairs:

- Bacterial ARISA forward: ITSF, 5'-GTC GTA ACA AGG TAG CCG TA-3' [11]
- Bacterial ARISA reverse: ITSReub, 5'-HEX-GCC AAG GCA TCC ACC-3' (Cardinale et al., 2004)
- Fungal ARISA forward: ITS1F, 5'-FAM-CTT GGT CAT TTA GAG GAA GTA A-3' [12]
- Fungal ARISA reverse: 3126T, 5'-ATA TGC TTA AGT TCA GCG GGT-3' [13]

PCR amplicons were resolved using an ABI 3130xl Genetic Analyzer (Applied Biosystems) at the University of Waikato DNA Sequencing Facility. All peaks between 100 and 1200 base pairs in length that constitute greater than 0.3 % of all peaks over 10 RFU (relative fluorescence unit) in each electropherogram were accepted as true peaks. The total number of true peaks in each sample was taken as a measure of taxon richness.

Soil respiration was measured by incubating 20 g (dry weight equivalent) samples of soils at 10°C for 26-28 days in an miniaturized respiromotric chambers [14] and the CO2 determined periodically by gas chemoatogrpahy (Varion90 GC fitted with a thermal conductivity detector) as described by [15].

Modelling approach and rationale

Our aim was to detect significant negative covariation between pairs of species potentially competing for resources. We had to test for non-random patterns in species co-occurrence and assess whether the negative correlation between species co-vary with other biotic and abiotic factors (Supplementary Figure 2). Models must also account for spatial autocorrelation, which can be caused by unmeasured population processes such as dispersal dynamics (e.g., source/sink dynamics and historical legacy) as well as unmeasured abiotic and biotic factors.

Models and analyses (additional information on GLMMs)

Binomial GLMMs were applied to the presence/absence distribution of species, which was modeled as a function of abiotic predictors such as topography and soil properties (moisture, pH) and biotic factors such as the biomass of other taxa. Given our *a-priori* hypothesis, the main aim of these models was to detect a negative effect of *Plectus* on *Scottnema* (and vice versa) and neutral effects of *Eudorylaimus* on *Plectus* and *Scottnema* but we needed to ensure that these negative effects were not indirectly caused by other abiotic and biotic covariates. We used GLMMs [16] to model spatial autocorrelation in the residuals using an exponential function, which was chosen after preliminary analyses. Specifically, to investigate spatial autocorrelation, we first ran a standard GLM that had the same structure of the GLMMs except for the spatial autocorrelation component. Residuals from these models were mapped and tested for autocorrelation using Moran's I statistics, autocorrelograms, and relevant tests (Dormann et al. 2007 and references therein). As we modelled the distribution of a given nematode species, the presence/absence distribution of each of the other two nematode species was used as a categorical predictor of the modelled species. GLMMs were run in R version 2.15.1 [18] using the package MASS.

Supplementary Results

Abiotic predictors

We split abiotic factors into topographical factors, which describe the geomorphology of the study area, and soil factors, which account for microscale variability in soil factors such as moisture, pH and salinity. Principal component analysis (PCA) of the correlation matrix of topographical variables (see Supplementary Figure 1, PCA_top) showed that at least three axes were necessary to account for more than 2/3 of variance (Supplementary

Figure 1, PCA.top): the first axis (PC1.top, 47 %) is an elevational gradient negatively correlated with temperature and positively correlated with slope and distance to the coast; the second axis (PC2.top, 20 %) basically accounts for the aspect of sampling points while the third axis (PC3.top, 17 %, not shown in Figure 3, PCA top) accounts for negative covariation between slope and the distance to the coast. Thus, in our linear models (see Table 1 and 2 in main text) we define PC1.top, PC2.top and PC3.top as Elevational gradient, Aspect, and Distance to coast gradient respectively.

Also in the case of the soil data matrix (Figure 3, PCA.soil), three PCA axes were necessary to meet the criterion of 2/3 of total variance: the first axis (PC1.soil, 33 %) accounts for a gradient in organic C and total N; the second axis (PC2.soil, 18 %) accounts for negative covariation between wetness or water and pH, while the third axis (PC3.soil, 18 %, not shown) is a gradient in electrical conductivity. In our linear models (see Table 1 and 2 in main text) we thus define PC1.soil, PC2.soil and PC3.soil as Organic matter gradient, Moisture gradient, and Salinity gradient respectively.

Biotic predictors

PCA of biotic variables (Figure 3, PCA.bio) identified the following three axes: the first one (PC1.bio, 33 %) accounts for a gradient separating sites with high microbial richness and high animal and microbial biomass from sites with low microbial richness and low animal and microbial biomass; the second one (PC2.bio, 18 %) accounts for negative covariation between, on the one hand, microbial biomass plus tardigrades and rotifers biomass, and on the other hand microbial richness and arthropod biomass; the third one (PC3.bio, 12 %; not shown) accounts for the fact that there were sites for which high biomass of mites and collembolans negatively correlated with the other biotic variables. . In our linear models (see Table 1 and 2 in main text) we thus define PC1.bio, PC2.bio and PC3.bio as

Microbial biomass gradient, Microbial richness gradient, and other fauna biomass gradient respectively.

Supplementary References

1. Freckman, D. W. & Virginia, R. A. Extraction of nematodes from Dry Valley Antarctic soils. *Polar Biol.* **13**, 483–487 (1993).
2. Timm, R. Antarctic soil and freshwater nematodes from the McMurdo Sound region. *Proc. Helminthol. Soc. Wash.* **38**, 42–52 (1971).
3. Andrássy, I. Nematodes in the sixth continent. *J. Nematode Morphol. Syst.* **1**, 107–186 (1998).
4. Andrássy, I. Eudorylaimus species (Nematoda: Dorylaimida) of continental Antarctica. *J. Nematode Morphol. Syst.* **11**, 11–1 (2008).
5. Boström, S., Holovachov, O. & Nadler, S. A. Description of Scottnema lindsayae Timm, 1971 (Rhabditida: Cephalobidae) from Taylor Valley, Antarctica and its phylogenetic relationship. *Polar Biol.* **34**, 1–12 (2011).
6. Caruso, T. & Bargagli, R. Assessing abundance and diversity patterns of soil microarthropod assemblages in northern Victoria Land (Antarctica). *Polar Biol.* **30**, 895–902 (2007).
7. Sinclair, B. J. & Stevens, M. I. Terrestrial microarthropods of Victoria Land and Queen Maud Mountains, Antarctica: implications of climate change. *Soil Biol. Biochem.* **38**, 3158–3170 (2006).
8. Stevens, M. I. & Hogg, I. D. Long-term isolation and recent range expansion from glacial refugia revealed for the endemic springtail Gomphiocephalus hodgsoni from Victoria Land, Antarctica. *Mol. Ecol.* **12**, 2357–2369 (2003).
9. Gressitt, J. L. *Entomology of Antarctica*. **10**, (American Geophysical Union, 1967).

10. Lee, C. K., Barbier, B. A., Bottos, E. M., McDonald, I. R. & Cary, S. C. The inter-valley soil comparative survey: the ecology of Dry Valley edaphic microbial communities. *Isme J.* **6**, 1046 (2012).

11. Cardinale, M. *et al.* Comparison of different primer sets for use in automated ribosomal intergenic spacer analysis of complex bacterial communities. *Appl. Environ. Microbiol.* **70**, 6147–6156 (2004).

12. Gardes, M. & Bruns, T. D. ITS primers with enhanced specificity for basidiomycetes-application to the identification of mycorrhizae and rusts. *Mol. Ecol.* **2**, 113–118 (1993).

13. Sequerra, J. *et al.* Taxonomic position and intraspecific variability of the nodule forming Penicillium nodositatum inferred from RFLP analysis of the ribosomal intergenic spacer and random amplified polymorphic DNA. *Mycol. Res.* **101**, 465–472 (1997).

14. Heilmann, B. & Beese, F. Miniaturized method to measure carbon dioxide production and biomass of soil microorganisms. *Soil Sci. Soc. Am. J.* **56**, 596–598 (1992).

15. Hopkins, D. & Ferguson, K. Substrate induced respiration in soil amended with different amino acid isomers. *Appl. Soil Ecol.* **1**, 75–81 (1994).

16. Venables, V. N. & Ripley, B. D. *Modern applied statistics with S*. (Springer-Verlag, 2002).

17. Dormann, F. C. *et al.* Methods to account for spatial autocorrelation in the analysis of species distributional data: a review. *Ecography* **30**, 609–628 (2007).

18. R Development Core Team. R: a language and environment for statistical computing. Vienna, Austria: R Foundation for Statistical Computing. *Url Http Wwwr-Proj.* (2009).
