# Supplementary material for: Nematodes in a polar desert reveal the relative role of biotic interactions in the coexistence of soil animals
Source: Commun Biol. 2019 Feb 15;2:63. doi: 10.1038/s42003-018-0260-y (PMC6377602; doi:10.1038/s42003-018-0260-y)
Supplement: Supplementary file 5 — Description of Additional Supplementary Files [file 42003_2018_260_MOESM5_ESM.docx]

**Description of additional supplementary items**

**Supplementary Data 1: Dataset analyzed in this paper.**

The dataset includes all the variables analysed in the paper, including the abundance of the nematodes, spatial coordinates, and the abiotic and biotic variables used as predictor of nematode species distribution.

**Supplementary Software 1: R code for multivariate analysis.**

This code can be run on Supplementary Data 1 to perform dbRDA and variance partitioning of the nematode multispecies distribution.

**Supplementary Software 2: R code for GLMMs.**

This code can be run on Supplementary Data 1 to perform GLMMs for each species.
